# Genomic signatures of the plateless phenotype in the threespine stickleback

**DOI:** 10.1002/ece3.2072

**Published:** 2016-04-06

**Authors:** Anna B. Mazzarella, Sanne Boessenkool, Kjartan Østbye, Leif Asbjørn Vøllestad, Emiliano Trucchi

**Affiliations:** ^1^ Department of BiosciencesCentre for Ecological and Evolutionary SynthesisUniversity of OsloPO Box 1066BlindernNorway; ^2^ Faculty of Applied Ecology and Agricultural SciencesHedmark University CollegeCampus EvenstadNo‐2480KoppangNorway; ^3^ Department of Botany and Biodiversity ResearchUniversity of ViennaRennweg 14A‐1030ViennaAustria

**Keywords:** Bayescan, *Gasterosteus aculeatus*, latent factor mixed models, lateral plates, RADseq

## Abstract

Understanding the genetic basis of traits involved in adaptive divergence and speciation is one of the most fundamental objectives in evolutionary biology. Toward that end, we look for signatures of extreme plate loss in the genome of freshwater threespine sticklebacks (*Gasterosteus aculeatus*). Plateless stickleback have been found in only a few lakes and streams across the world; they represent the far extreme of a phenotypic continuum (plate number) that has been studied for years, although plateless individuals have not yet been the subject of much investigation. We use a dense single nucleotide polymorphism dataset made using RADseq to study fish from three freshwater populations containing plateless and low plated individuals, as well as fish from full plated marine populations. Analyses were performed using FastStructure, sliding windows *F*_ST_, Bayescan and latent factor mixed models to search for genomic differences between the low plated and plateless phenotypes both within and among the three lakes. At least 18 genomic regions which may contribute to within‐morph plate number variation were detected in our low plated stickleback populations. We see no evidence of a selective sweep between low and plateless fish; rather reduction of plate number within the low plated morph seems to be polygenic.

## Introduction

A fundamental objective in evolutionary biology is to understand the genetic basis of traits involved in adaptive divergence and speciation (Ellegren 2008; McKay and Stinchcombe 2008). The study of adaptive radiations is a powerful way of investigating contemporary evolutionary processes, and the genetic changes underlying adaptation (Arendt and Reznick 2008; Conte et al. 2012). Ecological opportunity promoting differentiation, necessary for such adaptive radiations to occur, may happen through dispersal into new environments (Grant and Grant 2008; Yoder et al. 2010) or through range expansion (Parmesan et al. 1999). Such range expansions may occur due to climate change, or when new habitats become available through habitat modifications. When numerous freshwater habitats became accessible following the retreat of glaciers, various aquatic organisms rapidly invaded these new habitats. One such organism is the threespine stickleback (*Gasterosteus aculeatus*).

The threespine stickleback is a well‐known model organism for the study of adaptive evolution. Throughout the Northern Hemisphere this species colonized lakes and streams from its ancestral marine environment, following the glacial retreat at the end of the Pleistocene (Bell and Foster 1994). This resulted in repeated adaptation to freshwater, which has been associated with a range of morphological, behavioral and physiological changes, including the well‐described loss of lateral plates (Heuts 1947; Bell and Foster 1994; Colosimo et al. 2004). Marine fish have a full row of lateral bony plates on each side (30 or more plates, referred to as the full plated morph), while freshwater individuals have evolved such that only a part or in extreme cases none of this lateral armor remains (<10 plates; referred to as the low plated morph, Francis et al. 1985). A partially plated morph with intermediate numbers of plates is commonly found in brackish water, but also occasionally in freshwater or marine environments (Bell and Foster 1994). The loss of plates when colonizing freshwater has been observed to evolve rapidly, sometimes within just a single generation (Klepaker 1993; Bell 2001; Bell et al. 2004; Le Rouzic et al. 2011). Numerous theories have been presented to explain the functional mechanisms of armor loss, and how this may be related to changes in salinity or predation (Hagen and Gilbertson 1972; Moodie and Reimchen 1976; Giles 1983; Reimchen 1983; Kitano et al. 2008; Rennison et al. 2015). Nevertheless, the precise selective pressures are not yet clearly understood (Voje et al. 2013; MacColl and Aucott 2014; Mazzarella et al. 2015).

**Figure 1:**
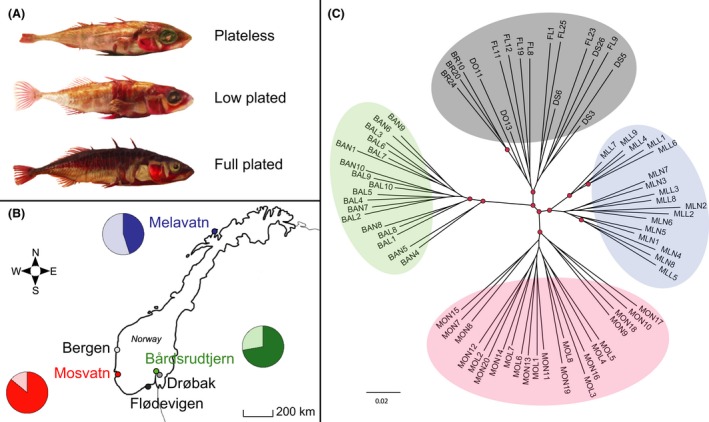
(A) Examples of a plateless, low plated and full plated threespine stickleback with bone stained with alizarin red for identification of lateral bony plates. (B) Map of study sites and pie charts showing proportion of low plated (dark color) and plateless (light color) fish in each population. Mosvatn is shown in red, Bårdsrudtjern green, Melavatn blue, and the marine populations (Drøbak, Flødevigen and Bergen) are gray. (C) Maximum‐Likelihood tree built using RAxML of all individuals showing genetic diversity, and population structure among all populations. Red circles indicate nodes with 100 bootstrap support. Populations are labeled by color overlay as per Figure B, individuals are labeled by Code and Fish # as listed in Table S1 (first two letters indicate population, third letter indicates plate morph for freshwater fish, as follows: L = low plated, N = plateless).

The gene *EDA* (*Ectodysplasin‐A*) has been identified as the major locus controlling plate morph variation (first described in Colosimo et al. 2004). The genotype at *EDA* predicts approximately 70% of the variation in plate morph (Colosimo et al. 2004), with low plated fish being homozygous for the low armor allele, full plated fish homozygous for the full armor allele, and partially plated fish being heterozygous (Fig. 1A). The prevailing hypothesis is that standing genetic variation in the marine populations enables the rapid plate loss as colonizers adapt to fresh water (Colosimo et al. 2005) as it is possible to find wild marine individuals that are heterozygous at the *EDA* gene (Barrett et al. 2008). Recently, a single nucleotide polymorphism (SNP) causing cis‐regulatory changes in the *EDA* enhancer has been identified, reducing the expression of developing plates in the low armor allele (O'Brown et al. 2015).

Although *EDA* controls plate morph to a large extent, it does not explain plate number variation within each morph (Colosimo et al. 2005). An extreme case of such variation constitutes the complete loss of plates (occasionally called the plateless “morph;” Fig. 1A). Although <10 plates is the traditional definition of “low plated,” from this point on in this article, when we refer to the “low plated” fish we refer to stickleback with 1–10 plates, whereas fish with 0 plates are “plateless.” In contrast with low plated stickleback, plateless stickleback are rare and have been reported from only a few lakes and streams in Norway, Scotland, British Columbia, and Alaska (Reimchen 1984; Klepaker 1995; Spence et al. 2013; MacColl and Aucott 2014). Interestingly, in some of these populations they are maintained in relatively high numbers (this study; Klepaker 1995). A complete lack of armor is thought to have significant fitness consequences, as the presence of lateral plates in the low plated morph stabilizes the stickleback's dorsal and pelvic spines and allows for greater retention of the anti‐predator functionality of this bony armor (Reimchen 1983): specifically, if the plates directly under the dorsal spines are severely reduced or missing entirely, the spines lose a large part of their function in predator defense. It is therefore expected that these specific plates would be preserved in low plated morphs (Reimchen 1983). By contrast, it has been proposed that a loss of plates may have the potential to improve agility (Andraso and Barron 1995; Andraso 1997; Bergstrom 2002). Taken together it appears likely that many differing selection regimes may act on plate number within the low plated morph, and on plateless fish in particular.

Here, we test if the extreme plateless phenotype is associated with genes/genomic regions using a dense SNP dataset created using the sequencing of restriction‐site associated DNA tags, or RADseq (Baird et al. 2008; Hohenlohe et al. 2010). We analyzed stickleback sampled from three freshwater populations containing plateless and low plated individuals. In addition, we compared these freshwater fish to marine stickleback to confirm known patterns of freshwater‐marine divergence. Several population genomic analyses and outlier analyses were applied to search for genomic differences between the low plated and plateless phenotypes both within and between the three lakes.

## Materials and Methods

### Sampling

Low plated and plateless threespine stickleback were sampled from three freshwater lakes (Melavatn, Mosvatn, and Bårdsrudtjern) along the Norwegian coastline in 2011 and 2012 (Fig. 1B). The lakes are connected to the oceans through more or less steep rivers. Downstream geneflow is possible, while contemporary upstream gene flow is improbable as stickleback are not strong swimmers. All three freshwater lakes contain populations of brown trout *Salmo trutta*, a well‐known piscivore. The geographic distance between each of the three lakes is more than 400 km, ensuring independent colonization events of threespine stickleback into each of the lakes. To confirm known patterns of freshwater‐marine divergence we additionally analyzed threespine stickleback sampled in three marine sites (Drøbak, Flødevigen, and Bergen; Fig. 1B). Lake samples were collected using baited minnow traps (Breder 1960). Marine samples were collected with handheld dip nets or with fine‐mesh seine nets (0.6–1.2 cm mesh size). Stickleback were stored directly in 96% ethanol.

For the genomic analyses, we selected 20–22 fish from each of our six populations (see Table S1 for more information on samples). From the marine populations the individuals were sampled randomly, while we collected at least 10 low plated and 10 plateless individuals from each freshwater lake. The exact number of plates was counted for all fish included in the latent factor mixed models (LFMM) analyses (see below, see also Table S1). In addition, 305 fish from Melavatn, 215 fish from Mosvatn, and 194 fish from Bårdsrudtjern were scored for presence/absence of plates in order to determine the proportion of low plated and plateless fish in each lake. To count plate number, sticklebacks were removed from individual storage containers and dried for approximately 5 min before one of us carefully counted all the lateral plates on both sides of the stickleback using a microscope.

### Laboratory methods

Genomic DNA was extracted using the DNeasy Tissue kit (Qiagen, Waltham, MA) following the manufacturer's guidelines. We created six RAD libraries of 24 individuals each, following the process outlined in Etter et al. (2011) with the following minor modifications: (1) approximately 100 ng of genomic DNA per sample was digested with the restriction enzyme *Pst*I (NEB); (2) each sample was ligated to a unique barcoded P1 adapter prior to pooling in a single library; (3) libraries were sheared by sonication on a Bioruptor (Diagenode) where the target size range fraction (300–500 bp) was achieved after six cycles of sonication; (4) after concentration to 25 *μ*L by DNA capture on magnetic beads (beads solution:DNA = 0.8:1), libraries were size selected by gel electrophoresis and manual excision; (5) capture on magnetic beads (beads solution:DNA = 0.8:1) was employed in all following purification steps (i.e., after blunt‐end repair, poly‐A tailing, P2 adapter ligation and library enrichment by PCR); (6) PCR amplification was performed in 8 × 12.5 *μ*L aliquots pooled after the amplification in order to reduce amplification bias; (7) DNA concentration of libraries was quantified by a fluorometric‐based method (Qubit^®^; Invitrogen) and molarity checked on an Agilent Bioanalyzer using an Agilent DNA 1000 Kit. A final volume of ca. 20 *μ*L per library with a DNA concentration of 20–25 ng/*μ*L was submitted for paired‐end 100 bp sequencing on the ILLUMINA HiSeq2000 sequencer at the Norwegian Sequencing Centre, University of Oslo. Libraries were sequenced in 12 lanes (each library on two lanes). Of the 144 individuals sequenced in these six libraries, 128 fish were included in the following downstream analyses, 68 freshwater (at least 20 from each population) and 60 marine (20 from each population) – 16 individuals from the total sequenced 144 were excluded due to poor sequencing quality.

### Sequence filtering and SNP calling

Raw sequence reads were demultiplexed using *Stacks* (Catchen et al. 2011). Entire read length of 95 bp was used, and those with correct or rescuable barcodes, high sequencing quality according to the Illumina quality scores (e.g., when the average quality score per base in any window of 15% of the read length dropped below 10, the read was discarded), and unambiguous RAD sites were retained, according to the default Stacks protocol. Reads that passed the first filtering were aligned to the threespine stickleback genome assembly (Ensembl; database release 72) using GSNAP (Wu and Nacu 2010), allowing unique alignments with up to five mismatches and two indels per read. We did not allow for terminal alignments, which prevents soft masking of large fractions of either sequence end (Catchen et al. 2013). Aligned reads were analyzed in *Stacks*, resulting in a consensus sequence for each locus. Loci with fewer than five reads for an individual were discarded from that individual. Using *Stacks*, we called SNPs and identified individual genotypes using a maximum‐likelihood (ML) statistical model.

The catalog of loci created by Stacks was exported, checked and filtered using custom *python* scripts (in‐house scripts available upon request). Filtering out individuals with more than 75% missing loci resulted in 107,240 loci in 74 individuals with an average of 35% missing data per locus. Plotting the positions of the SNPs along the sequence across all the loci, we observed an unexpected increase in the occurrence of SNPs in the last three base pairs. As SNPs should be evenly distributed along the sequences, we identified this accumulation as an artifact of the SNP calling process. Trimming the reads to a shorter length before the SNP calling didn't alleviate the problem; we therefore removed SNPs found in the last three base pairs of the sequence. We further filtered for a maximum of 65% heterozygosity. We set this arbitrary cut‐off to reduce the risk of including paralogs (with alleles coming from different duplicated loci in the genome) in our dataset. GC content was normally distributed in our loci with an average value of 0.47, and SNPs were distributed equally across the length of the read until 92 bp (Figure S1) This final filtering resulted in a dataset containing 92,979 loci genotyped in 74 samples that were used for the subsequent analyses, with the exception of the pairwise *F*_ST_ analyses that was run using the script *populations* within *Stacks,* applying a different filtering strategy to our initial catalog (see below).

### Population structure analyses

Overall genetic diversity and structure across all studied populations was assessed using RAxML (Stamatakis et al. 2008), a program for ML phylogenetic analysis of large datasets. All variable sites were extracted from all loci in the dataset and concatenated coding heterozygous sites as ambiguities. A rapid bootstrap analysis (100 replicates) and search for the best‐score ML tree (‐f a option) was set. The nucleotide substitution model was specified as GTRCATX where a General Time Reversible model is coupled with a fast model for optimization of heterogeneity of evolutionary rates across sites (CAT model). The General Time Reversible model is the only option in RAxML as it has been recognized as the best‐fitting model in analyses of large SNPs dataset. Through the “X” option, a ML estimate of base frequencies was also set. The CAT model for rate heterogeneity among sites has proven to be as accurate as a Gamma model and much faster when applied to very large genomic dataset, as in our case (Stamatakis 2006). Results were visualized using *Figtree* (Rambaut and Drummond 2012).

FastStructure (Raj et al. 2014) was used to explore the overall structure among the two plate morphs within each freshwater population testing a single group versus a two‐groups structure (*k* = 1–2). FastStructure performs inference for the simplest, independent‐loci, admixture model as an implementation of the software Structure, specifically suited for analyzing large datasets of bi‐allelic loci such as SNPs. It uses a variational Bayesian framework for inferring the structure in the population and heuristic scores to identify strong population structure in the data. As only independent biallelic loci are allowed, we selected one SNP at random from each RAD locus. In order to speed up the computations, the dataset was reduced to a random selection of 4850 bi‐allelic loci that were considered as suitable to provide evidence of any signature of genome‐wide population structure in the data. We tested a flat beta prior distribution over population‐specific allele frequencies at each locus (linear prior) using a range of *k* values (i.e., number of groups) from 1 to 2. The script *choose.py* included in the fastStructure package provides two estimates of the best *k*: one that maximizes the marginal likelihood, and a second estimate that best explains even very weak structure in the data. Both values for *k* were stored and the analysis repeated 100 times. We further ran fastStructure on all samples, including the three marine populations, exploring *k* values between 2 and 8. As before, 100 independent replicates including the structure.py script (linear prior) on the selected values for *k* and the choose.py script were run and results checked for convergence.

### Outlier loci analyses

Using the *populations* program within *Stacks* we calculated kernel‐smoothed *F*_ST_ for all pairwise freshwater‐marine population comparisons as well as low plated versus plateless phenotype comparisons per population. For these analyses we started from the catalog in Stacks containing 128 individuals and only kept loci present in more than 20% of the individuals within a population, and found in at least two populations. We used a sliding window size of 150,000 bp (nonoverlapping) for the kernel smoothing, and filtered out any alleles with a frequency of <0.015. The smoothed *F*_ST_ was plotted against physical location across each chromosome for each within‐lake morph pair and for each freshwater versus marine population comparison using R v.2.10.1 (R Developmental Core Team, Vienna, Austria). We visually layered all of our pairwise *F*_ST_ comparisons to detect areas of high *F*_ST_ that converge across the comparisons.

Two additional tests for detecting outlier loci were performed using the set of 92,979 loci resulting from the data filtering described above under “Sequence filtering and SNP calling.” The fully Bayesian approach developed by Foll and Gaggiotti (2008) and implemented in the software Bayescan was used to estimate the probability that each specific locus is subject to selection in the comparison between low plated and plateless morphs. Bayescan searches for loci exhibiting extreme *F*_ST_ values that are then interpreted as signatures of local adaptation. It incorporates the uncertainty due to small sample size in the inference and it is therefore suited for the comparison between the two morphs within each lake separately. Bayescan was run on each freshwater lake sample separately to search for outliers between the plateless and lowplated fish. For each analysis, we ran two long chains of 50,000 iterations using prior odds of 100 and assessed the statistical significance of a locus being an outlier by the use of *q*‐values. As we expected to find outlier loci differentiating all marine fish versus all freshwater fish, we ran an additional analysis between these two groups to confirm the presence of these expected outliers.

For the third outlier analysis we used the program “latent factor mixed models” or LFMM (Frichot et al. 2013) to detect relationships between allele frequencies and actual plate count number in the freshwater populations, while taking into account population structure. This is a very general and flexible model based on the covariance between allelic frequencies and an environmental gradient, and provides an alternative approach to the extreme *F*_ST_‐based search implemented in Bayescan. This method is particularly suited to detect small‐effect loci that are contributing to a chosen biological gradient. We did not use an environmental gradient but instead used as input a meristic trait, plate count, under the assumption that a phenotypic gradient should show a similar pattern to an environmental gradient so long as the trait in question is under selection. We therefore analyzed all freshwater individuals for which the plates had been counted (54 individuals), using the number of plates as the trait in question (range: 0–8, see Table S1). As the number of latent factors should reflect the expected genetic structure in the sample and then the number of populations identified by the structure analysis (i.e., the three lakes), we set the latent factors to 3. In this case, one random SNP per locus was selected and invariant loci across analyzed individuals were carefully excluded returning an input dataset of 57,999 loci. Five replicates were checked for convergence. When using a cutoff for the −log_10_(*P*‐value) of 5, ca. 600 putative outliers were detected in each run. To focus only on the most extreme outliers we applied two independent criteria. First, loci showing a −log_10_(*P*‐value) above 7 were grouped along the chromosomes using a window size of 500,000 bp and only windows containing three (or more) significant loci were retained for further investigation. Additionally, the top 10 outlier loci (1.5% of the 600 loci passing the initial threshold) consistently returned by all five replicated runs (−log_10_(*P*‐value) above 16) were directly retained for further investigation. Significant loci were investigated to ensure that missing data were not influencing the categorization of these regions as outliers in this analysis (Table S2), and subsequently annotated using the stickleback genome reference in the Ensemble database (Table 1). Gene ontology terms, or GO terms for the nearest genes were also recorded (Table S3).

**Table 1:** Outlier loci identified using LFMM. Chr# and Position refer to the exact location of the outliers in the genome (Ensembl, database release 72). −log_10_(*p*‐score) and *p*‐score are output from LFMM that determine the strength of the association with the trait in question (here, plate number). The STN marker related to plate number described in Colosimo et al. (2004) is bold and listed under “Nearest gene.”

Outlier number	Chr. #	Position	−log_10_(*P*‐value)	*P*‐value	Nearest gene(s)	Position
LFMM top 0.1% individual outliers						
Outlier 1	1	18,831,918	17.2831	5.21066e‐18	bcas3‐202	Group I: 18,784,504–18,879,930
					bcas3‐203	Group I: 18,840,087–18,879,930
Outlier 2	2	17,346,118	21.5028	3.14177e‐22	fto	Group II: 17,300,817–17,331,950
					irx3a	Group II: 17,179,737–17,181,705
Outlier 3	6	3,203,562	17.0184	9.58503e‐18	wdr11	Group VI: 3,165,689–3,200,700
					ppapdc1a	Group VI: 3,252,638–3,265,797
Outlier 4	8	16,417,215	16.7516	1.77189e‐17	creb3l3a‐201	Group VIII: 16,297,685–16,302,922
					rxfp3.2b	Group VIII: 16,288,935–16,289,999
Outlier 5	10	6,215,621	18.0032	9.92676e‐19	SLC45A4A	Group X: 6,207,305–6,226,049
					DENND3	Group X: 6,176,151–6,189,656
					**STN211**	Group X: 6,169,732
Outlier 6	10	1,194,119	17.7348	1.84157e‐18	VPS13B	
Outlier 7	14	9,371,286	16.8383	1.45111e‐17	palm2	Group XIV: 9,332,963–9,357,146
Outlier 8	14	14,631,941	20.6216	2.38989e‐21	ier5l	Group XIV: 14,632,965–14,633,933
Outlier 9	20	15,901,231	18.2046	6.24309e‐19	rpz	Group XX: 15,894,758–15,898,490
					rpz3	Group XX: 15,902,147–15,904,180
Outlier 10	21	3,933,665	18.6495	2.24109e‐19	eef1e1	Group XXI: 3,900,261–3,905,214
					slc35b3	Group XXI: 3,947,748–3,952,816
LFMM outlier regions from binning						
Outlier 11	2	9,006,584	8.2813	5.23E‐009	cilp‐201	Group II: 9,005,752–9,010,198
		9,184,158	8.33758	4.60E‐009	tjp1a‐201	Group II: 9,116,332–9,139,014
		9,270,579	9.11876	7.61E‐010	ENSGACG00000015593	Group II: 9,283,463–9,292,122
Outlier 12	3	12,301,942	7.01227	9.72E‐008	loxl5a‐202	Group III: 12,295,563–12,298,888
		12,314,917	10.2857	5.18E‐011	ENSGACG00000016917	Group III: 12,306,094–12,307,625
		12,320,008	8.43104	3.71E‐009	Myo9b‐201	Group III: 12,322,870–12,341,345
Outlier 13	7	20,035,506	10.9751	1.06E‐011	stard13a‐201	Group VII: 20,033,223–20,046,170
		20,104,697	7.70877	1.96E‐008	nbeaa‐201	Group VII: 20,054,776–20,129,917
		20,281,583	12.4319	3.70E‐013	nbeaa‐202	Group VII: 20,107,491–20,129,917
Outlier 14	14	8,046,890	8.01584	9.64E‐009	bcr‐201	Group XIV: 8,099,548–8,156,144
		8,289,650	13.7757	1.68E‐014	RIMBP2 (2 of 2)	Group XIV: 8,224,105–8,236,955
		8,309,794	7.40911	0.000000039	si:dkey‐112m2.1	Group XIV: 8,297,103–8,367,291
Outlier 15	14	14,545,075	8.38684	4.10E‐009	lmo4a‐201	Group XIV: 14,567,743–14,574,881
		14,629,262	12.3632	4.33E‐013	ntmt1‐201	Group XIV: 14,607,347–14,610,905
		14,631,941	20.6216	2.39E‐021	ier5l‐201	Group XIV: 14,632,965–14,633,933
		14,653,233	8.63007	2.34E‐009	crata‐201	Group XIV: 14,653,383–14,665,803
Outlier 16	15	9,174,098	8.43889	3.64E‐009	dph6‐201	Group XV: 9,094,722–9,143,933
		9,254,647	7.08545	8.21E‐008	C15orf41‐201	Group XV: 9,224,970–9,273,325
		9,497,967	8.45721	3.49E‐009	ptpn21‐201	Group XV: 9,490,435–9,501,192
Outlier 17	16	5,673,800	8.55503	2.79E‐009	cx43.4‐201	Group XVI: 5,613,907–5,615,139
		5,793,565	7.65413	2.22E‐008	gulp1a‐201	Group XVI: 5,748,920–5,803,548
		5,949,642	14.8078	1.56E‐015	igfbp5b‐201	Group XVI: 5,921,243–5,930,904
Outlier 18	20	7,053,842	8.18001	6.61E‐009	SLC6A3‐201	Group XX: 7,033,258–7,043,379
		7,065,859	9.37619	4.21E‐010	neurensin 1	Group XX: 7,068,384–7,072,757
		7,066,256	7.82991	1.48E‐008		

## Results

The proportion of plateless fish was 14%, 28%, and 55% in the lakes Mosvatn, Bårdsrudtjern, and Melavatn respectively (Fig. 1B). The other fish in the lakes were low plated (range: 2–8; Table S1), while all fish in the marine populations were full plated.

A tree‐like representation of the structure and connectivity between the populations made using RAxML supported four separate groups, where each lake was represented by a single cluster, and the fourth cluster included all fish from the three marine populations (Fig. 1C). No detectable structure was found between the Oslo fjord and the West coast marine fish, and each freshwater lake branched out individually. None of the freshwater populations was more closely related to the marine population from a nearby locality than all the marine populations among themselves.

**Figure 2:**
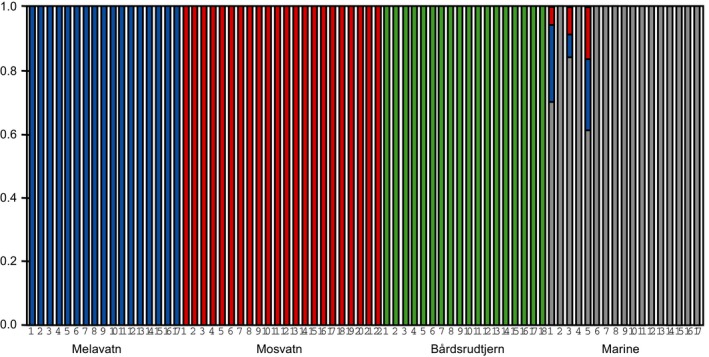
Genetic clustering analysis using FastStructure on all populations with *K* = 4. Individuals are ordered per population and population identity is shown along the bottom. Colors represent the different clusters and correspond to the four population colors used in Fig. 1B and C.

Using the Bayesian clustering algorithm as implemented in fastStructure, the overall structure of the data was best explained with four clusters (*K* = 4), consistent with the results from the ML analysis (Fig. 2). When the lakes were each analyzed individually, no internal structure was discovered and a single panmictic population was confirmed in Mosvatn and in Bårdrudstjern. In Melavatn, there was slightly higher support for *K* = 2 than *K* = 1 (Figure S2). However, the two clusters in this population did not segregate according to plate morph (plateless vs. low plate).

**Figure 3:**
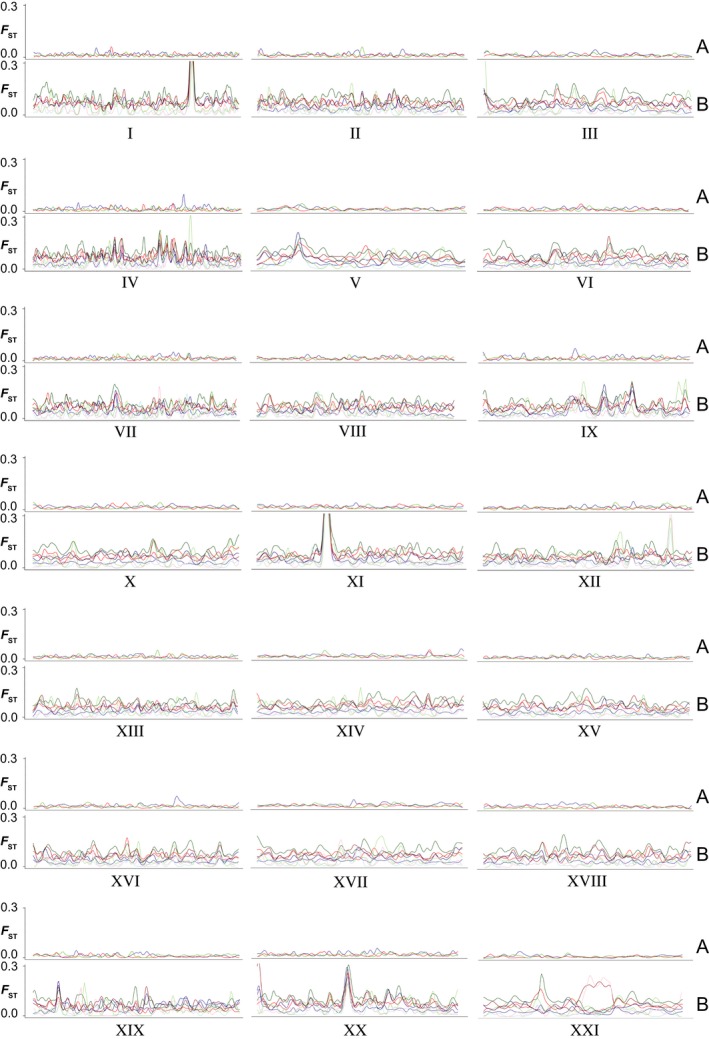
Kernel‐smoothed *F*_ST_ plotted against physical location on each chromosome (labeled by chromosome number). *F*_ST_ range is from 0.0 to 0.3. Top rows (A) show the comparison between low plated fish and plateless fish for each lake independently. Bottom rows (B) show the comparison between each freshwater population and each marine population (nine pairwise comparisons). Freshwater: Mosvatn is shown in red, Bårdsrudtjern green, Melavatn blue. Marine: Flødevigen comparisons are dark, Drøbak medium, and Bergen comparisons shown with light colors. For example, the light red line is Mosvatn v. Bergen. Comparing the top and bottom rows, it is clear that there is a strong pattern in the bottom rows (B), showing the differentiation between the freshwater and marine populations, while there is no differentiation between the low plated and plateless individuals within lakes, as is seen in the top rows (A).

*F*_ST_ analyses between each pair of fresh‐ and salt water populations (Fig. 3) showed a pattern of divergence that is very similar to that seen in Jones et al. (2012b) (e.g., the same areas of differentiation and peaks of similar amplitude), where the difference between freshwater and marine populations across the globe was analyzed using full genome sequencing data. For example, inversions on Chr I and XI found by Jones et al. are seen clearly. We also see a large inversion on Chr XXI in one of our three freshwater populations (Mosvatn) which is consistent with a large inversion found in some, but not all freshwater populations by Jones et al. (2005). *EDA* is also distinguishable on Chr IV with the series of high peaks in the graphs, and another large peak on Chr XX is within a previously described region of high differentiation seen in Jones et al. (2005). Lower peaks on Chr V, IX, and XIX are also consistent with regions found in Jones' analysis. Our *F*_ST_ analysis comparing low and plateless fish showed no outlying peaks in *F*_ST_, both when the freshwater populations were pooled together (all plateless fish compared with all low plated fish, not shown) and when each population was analyzed separately (Fig. 3).

**Figure 4:**
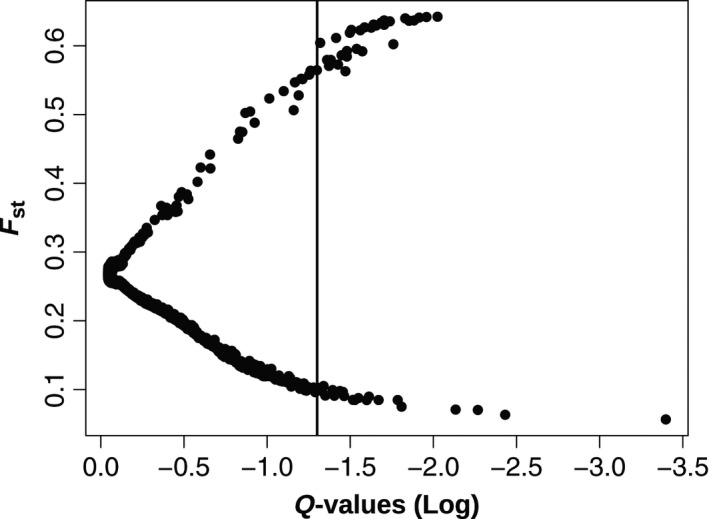
Visualization of the outlier analysis using Bayescan, all freshwater populations compared to all marine populations. The line denotes a significance threshold with False Discovery Rate as 0.05; loci to the right of the line are significant outliers.

No outliers were identified between plateless and low plated fish within any of the three lakes using Bayescan (results not shown). By contrast, many outliers were detected when Bayescan was run to compare the freshwater and the marine fish (Fig. 4).

**Figure 5:**
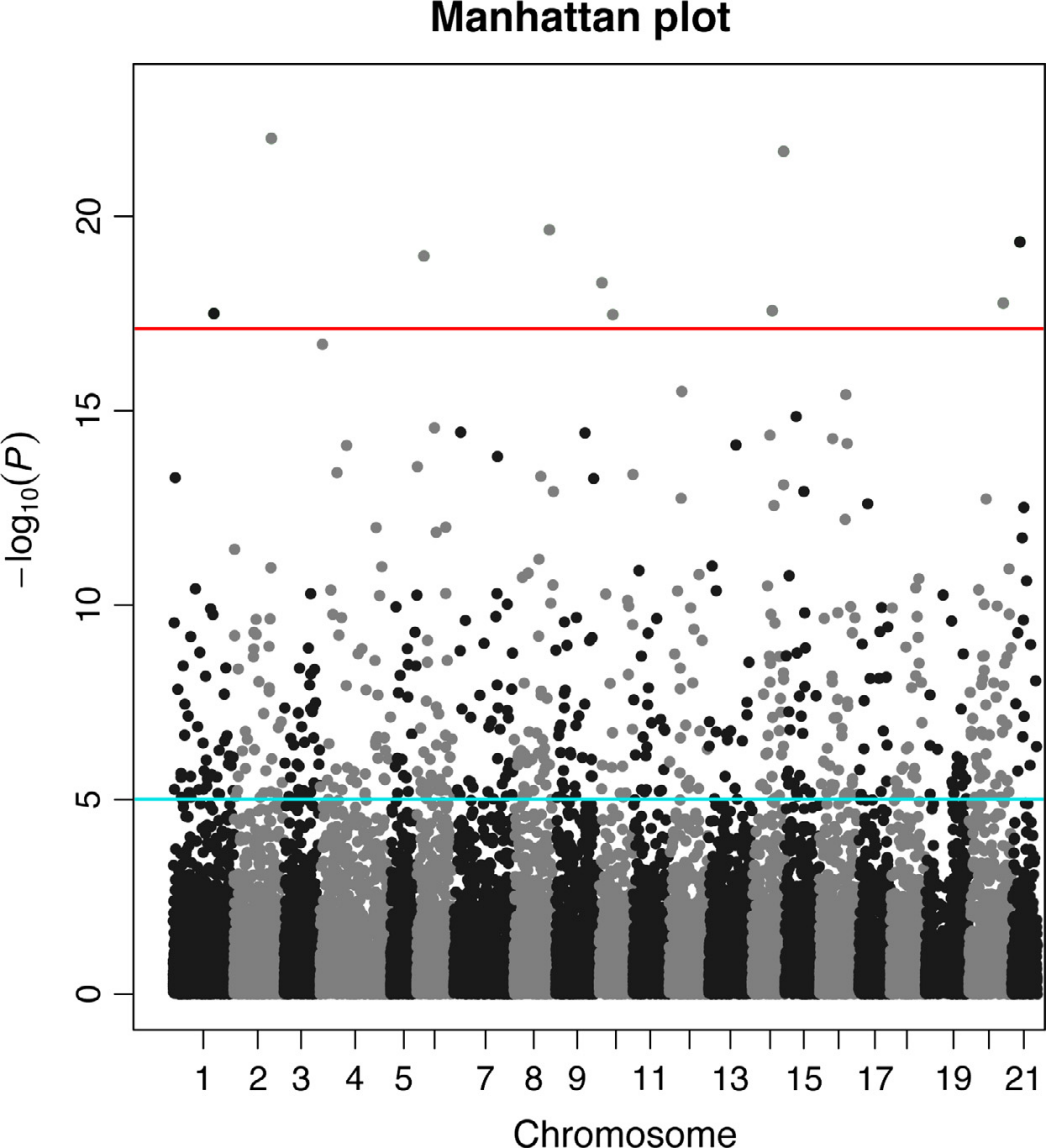
Manhattan plot produced by LFMM showing −log_10_(*P*‐value)s for all loci, indicating the strength of their covariation with plate number. Recommended cutoff is shown by the cyan line at 5, our more stringent cutoff, that is, the top 10 outliers, is shown with the red line.

Using LFMM, we retrieved 10 outlying loci (Fig. 5, Table 1) and eight regions in the genome with small clusters of significant outliers, giving a total of 18 outliers/outlying regions (Table 1). None of the outliers lie inside the coding region of identified genes, however, outlier 5 on chromosome X (at base position 6,215,621) is close to (<46 kbp away from) a previously described microsatellite (STN 211 at position 6,169,732) that was shown to modify plate number within the low plated morph (Colosimo et al. 2004).

The nearest genes for each significant outlier or outlier region were annotated (Table 1), and Gene Ontology keywords (“GO keywords”) were categorized to see the most abundant keywords. We found that “protein binding” was the most abundant, with “membrane component” and “DNA binding” being the next most abundant key words associated with the function of the proteins coded by these genes (Table S3).

## Discussion

### Robustness and limitations of the method

Genome‐wide association studies (GWAS) linking phenotypic traits to their underlying genetic basis have been widely used in model and nonmodel species, although their power and reproducibility have been often questioned (Ward and Kellis 2012). In the present study, we validated our genomic dataset by testing for well‐known patterns of differentiation between marine and freshwater stickleback before using this dataset to look for genetic differentiation between low plated and plateless freshwater stickleback. As the risk of false discovery is always inherent in any GWAS, we focused only on the most extreme outliers in the statistical tests applied (see “Materials and Methods”) and we excluded that a low sample size at these loci could have biased the results (Table S2). Although this practice may mitigate the risk of false or spurious correlations, GWAS outliers have to be considered as merely loci of interest that have to be confirmed in more in‐depth investigation on a larger number of individuals.

The use of PstI restriction enzyme in this study resulted in one tag (e.g., ~100 bp sequence) every ~10 kbp after filtering, which is high coverage for RADseq, but less coverage than typically achieved with whole‐genome sequencing. Yet, using sliding window *F*_ST_, we replicate the pattern of genomic divergence found in Jones et al. (2012b) when we compare fresh‐ and marine populations, even using a simpler analytical method of analysis and a relaxed filter for missing data. This indicates that our data contains sufficient information for comparing regions of high divergence, and indicates that relatively simple analytical methods can be used to discern those patterns of differentiation. Our fastStructure and RAxML results for all the populations also show a pattern that is typical of stickleback populations across the world (Hohenlohe et al. 2010; Jones et al. 2012a, 2012b; Feulner et al. 2015), with freshwater populations branching out independently and geographically close marine populations grouping together.

In addition to sliding window *F*_ST_ analysis, we used two other methods to detect outliers, one based on population differentiation (Bayescan) and the other based on the covariance between allelic frequencies and lateral plate counts (LFMM). LFMM has proven to be more effective in detecting the signature of polygenic selection (small‐effect loci), although it is characterized by a relatively high false discovery rate (de Villemereuil et al. 2014), so we should be aware that some of the loci detected might be false positives. On the plus side, LFMM should not be biased by any underlying geographic structure in the sample, as the structure is taken into account through a selected number of latent factors. Bayescan is less suited for detecting polygenic selection, but it is more conservative, resulting in a much lower false discovery rate. In our case, LFMM suggested a high number of loci correlated with plate number, while Bayescan found no outliers. We applied strict filtering on the outliers resulting from the LFMM analysis before annotating them on the stickleback reference genome (see “Materials and Methods”) to minimize the likelihood of false positives. In order to investigate as many loci as possible, we included in both analyses loci with up to 56% missing data. Latent factor models are designed to handle large amount of missing data well, and Bayescan has the advantage that it incorporates the uncertainty in the estimates of allelic frequencies into the estimation of all other parameters (Foll and Gaggiotti 2008). Overall, detecting the effects of selection on multiple loci of small effect is one of the most difficult tasks for genome scan methods, and a powerful approach specifically tailored to this situation has not yet been developed. In a previous study, LFMM method was demonstrated to be more effective than Bayescan to detect such loci (de Villemereuil et al. 2014), and our results further support this finding. The use of actual plate count data in LFMM analysis rather than a binary categorization of low plated compared to plateless fish, as in the Bayescan analysis, could be one reason why LFMM was able to detect these outliers while our other method was not.

### The genetic basis of platelessness in sticklebacks

Using a dense SNP dataset with sufficient resolution to depict the genomic divergence previously found between marine and freshwater populations of stickleback (Jones et al. 2012b), we found at least 18 outlying loci/genomic regions which may contribute to within‐morph plate number in our low plated stickleback populations. By contrast, we see no evidence of a selective sweep of a single gene or a few genes of large effect when contrasting low and plateless fish using sliding window *F*_ST_. Outlier 5, one of our top scoring outliers, is quite close (46 kb) to STN 211, a previously described microsatellite that is linked to a plate number modifier (Colosimo et al. 2004). Our results indicate that platelessness may be regulated by multiple loci of small‐medium effect (i.e., polygenic trait). This study, then, joins the body of work demonstrating that finding genes with a major effect on a phenotype is uncommon, and instead polygenic traits are prevalent (Rockman 2012). Indeed, it is likely that for some traits, many hundreds of genetic variants are contributing to them, and that selection acts simultaneously on variants at many different loci (polygenic adaptation) that each have a small effect on the trait in question (Pritchard et al. 2010). Retrieving the many relevant genomic loci contributing to a certain trait under selection using classic GWASs is a challenging task (Yang et al. 2010). While hard sweeps (sweeps of advantageous new mutations) should leave an easily detectable signal in the genome by spreading the effect of selection on a large neighboring region, soft sweeps (sweeps from slightly advantageous mutations, or variants present as previously neutral standing variation) leave a signal showing little contrast with the neutral background variation (Teshima et al. 2006).

Complexes of multiple genes in interaction with the environment are often involved in shaping phenotypic traits (West‐Eberhard 1989; Barry 2008). Plate phenotypes are clearly influenced strongly by *EDA* genotype (Colosimo et al. 2005), but are also modified by other genes (Colosimo et al. 2004). Additional modulators such as epigenetic modifications or phenotypic plasticity may also be important; indeed, it has been shown that phenotypic plasticity has a strong effect on stickleback morphology in response to different environmental cues (Day et al. 1994; Wund et al. 2008, 2012; Svanbäck and Schluter 2012; Lucek et al. 2014; Mazzarella et al. 2015), including stickleback plate number plastically responding to a change in salinity (Hansson et al. 2016). As such, it is possible that these or other environmental cues could be modulating the number of plates within plate morph, possibly along a reaction norm that is established by genotype (*EDA* or otherwise) (Schlichting and Pigliucci 1998). Epigenetic modifications are another possibility for moderating plate number within plate morph in the stickleback, and recent evidence has found multiple differentially methylated regions between low plated and full plated stickleback (Smith et al. 2015; Trucchi et al. 2016). Common garden experiments in which plate number is tracked through multiple generations under controlled conditions could improve our understanding of the effects of plasticity, maternal/paternal effects, and heritability on plate number within the low plated morph.

Plate number evolution in the stickleback system is often used as a clear example of parallel evolution by natural selection acting in response to known selection pressures. Yet the debate continues about what the selection pressures acting on plate number actually are, why this evolution is so parallel and so rapid, and how much variation is explained by *EDA* genotype (Spence et al. 2013; Voje et al. 2013; MacColl and Aucott 2014). Beyond the selection pressures causing plate loss, other questions remain about the plateless phenotype. Curiously, plateless stickleback are found in very few lakes and streams across the world, and always are sympatric with low plated fish, meaning that entire populations do not seem to evolve to complete platelessness (Reimchen 1984; Klepaker 1995; Spence et al. 2013). Many disparate selective pressures may act on plate number: it seems advantageous to have reduced bony armor in freshwater, this is clear from the global pattern of plate reduction in fresh water, however, it has been hypothesized that a complete loss of the lateral plates is negatively selected because complete plate loss leads to the complete loss of function of the dorsal spine, and thus a total lack of defense (Reimchen 1983). There is some evidence supporting this hypothesis including the observation that stickleback with fewer than five lateral plates have a reduced ability to survive attacks from predatory fish (Reimchen 1992). Alternatively, an extreme loss of armor might be advantageous if it gives the stickleback a better chance to avoid a possible attack in the first place, as suggested by the strong negative relationship found between armor robustness and startle performance (Andraso and Barron 1995; Andraso 1997; Bergstrom 2002), and the clear link between “faster starts” and the ability to evade predation (Walker et al. 2005). Within the low plated morph, it is even possible that there could be both positive selection both on a plate number of 0 (those being the fish best able to *evade* attacks), and also the very top of the plate number distribution (those being the fish best able to *survive* attacks). If the fish do not mate assortatively, this could preserve the pattern we see of lakes with a wide range of plate number within the low plated morph, including plateless individuals.

The absence of clear genetic differentiation between plateless stickleback and those that are low plated – as shown in our study – suggests plateless fish should not be considered a fourth plate morph category (in addition to the low, partial and full plated morphs), but we may continue to place them within the low plated morph. Nevertheless, it remains that plateless fish are a unique phenomenon and a better understanding of the variation in selection pressures on completely plateless versus low‐plated fish is needed to fully understand why plateless fish can be maintained in relatively high proportions in some populations while they are absent in the majority of the stickleback distribution.

## Data Accessibility

Raw reads have been uploaded to the NCBI Sequence Read Archive at this link: http://www.ncbi.nlm.nih.gov/bioproject/314185.

## Conflict of Interest

None declared.

## Supporting information


**Appendix S1.**
**Figure S1.** (A) Average number of SNPs by position across loci for all reads in all libraries showing a very slight, nonsignificant increase after base 92. (B) Mean GC content across loci, showing a normal distribution.
**Figure S2.** Genetic clustering analysis using FastStructure on the Melavatn population with *K* = 2.
**Table S1.** Sample information: Fish # refers to the number of the fish within a Code.
**Table S2.** Comma Separated Values containing 34 outlier loci (Table 1) and their genotype for all freshwater individuals included in the LFMM analysis.
**Table S3.** Gene ontology keywords ranked by abundance for the nearest gene(s) listed in Table 1.

## References

[ece32072-bib-0001] Andraso, G. M. 1997 A comparison of startle response in two morphs of the brook stickleback (*Culaea inconstans*): further evidence for a trade‐off between defensive morphology and swimming ability. Evol. Ecol. 11:83–90.

[ece32072-bib-0002] Andraso, G. M. , and J. N. Barron . 1995 Evidence for a trade‐off between defensive morphology and startle‐response performance in the brook stickleback (*Culaea inconstans*). Can. J. Zool. 73:1147–1153.

[ece32072-bib-0003] Arendt, J. , and D. Reznick . 2008 Convergence and parallelism reconsidered: what have we learned about the genetics of adaptation? Trends Ecol. Evol. 23:26–32. 18022278 10.1016/j.tree.2007.09.011

[ece32072-bib-0004] Baird, N. A. , P. D. Etter , T. S. Atwood , M. C. Currey , A. L. Shiver , Z. A. Lewis , et al. 2008 Rapid SNP discovery and genetic mapping using sequenced RAD markers. PLoS One 3:1–7. 10.1371/journal.pone.0003376 PMC2557064 18852878

[ece32072-bib-0005] Barrett, R. D. H. , S. M. Rogers , and D. Schluter . 2008 Natural selection on a major armor gene in threespine stickleback. Science 322:255–257. 18755942 10.1126/science.1159978

[ece32072-bib-0006] Barry, P. 2008 No gene is an island: even as biologists catalog the discrete parts of life forms, an emerging picture reveals that life's functions arise from interconnectedness. Science News 174:22–26.

[ece32072-bib-0007] Bell, M. A. 2001 Lateral plate evolution in the threespine stickleback: getting nowhere fast. Genetica 112(1):445–461. 11838781

[ece32072-bib-0008] Bell M. A., and Foster S. A., eds. 1994 The evolutionary biology of the threespine stickleback. Oxford Univ. Press, New York.

[ece32072-bib-0009] Bell, M. A. , W. E. Aguirre , and N. J. Buck . 2004 Twelve years of contemporary armor evolution in a threespine stickleback population. Evolution 58:814–824. 15154557 10.1111/j.0014-3820.2004.tb00414.x

[ece32072-bib-0010] Bergstrom, C. A. 2002 Fast‐start swimming performance and reduction in lateral plate number in threespine stickleback. Can. J. Zool. 80:207–213.

[ece32072-bib-0011] Breder, C. N. 1960 Design for a fry trap. Hydrobiologica 45:155–160.

[ece32072-bib-0012] Catchen, J. M. , A. Amores , P. Hohenlohe , W. Cresko , and J. H. Postlethwait . 2011 Stacks: building and genotyping loci de novo from short‐read sequences. G3 (Bethesda) 1:171–182. 22384329 10.1534/g3.111.000240 PMC3276136

[ece32072-bib-0013] Catchen, J. , P. A. Hohenlohe , S. Bassham , A. Amores , and W. A. Cresko . 2013 Stacks: an analysis tool set for population genomics. Mol. Ecol. 22:3124–3140. 23701397 10.1111/mec.12354 PMC3936987

[ece32072-bib-0014] Colosimo, P. F. , C. L. Peichel , K. Nereng , B. K. Blackman , M. D. Shapiro , D. Schluter , et al. 2004 The genetic architecture of parallel armor plate reduction in threespine sticklebacks. PLoS Biol. 2:635–641. 10.1371/journal.pbio.0020109 PMC385219 15069472

[ece32072-bib-0015] Colosimo, P. F. , K. E. Hosemann , S. Balabhadra , G. Villarreal Jr. , M. Dickson , J. Grimwood , et al. 2005 Widespread parallel evolution in sticklebacks by repeated fixation of ectodysplasin alleles. Science 307:1928–1933. 15790847 10.1126/science.1107239

[ece32072-bib-0016] Conte, G. L. , M. E. Arnegard , C. L. Peichel , and D. Schluter . 2012 The probability of genetic parallelism and convergence in natural populations. Proc. Biol. Sci. 279:5039–5047. 23075840 10.1098/rspb.2012.2146 PMC3497250

[ece32072-bib-0017] Day, T. , J. Pritchard , and D. Schluter . 1994 A comparison of two sticklebacks. Evolution 48:1723–1734. 10.1111/j.1558-5646.1994.tb02208.x 28568405

[ece32072-bib-0018] Ellegren, H. 2008 Comparative genomics and the study of evolution by natural selection. Mol. Ecol. 17:4586–4596. 19140982 10.1111/j.1365-294X.2008.03954.x

[ece32072-bib-0019] Etter, P. D. , P. A. Hohenlohe , E. A. Johnson , W. A. Cresko , and S. Bassham . 2011 SNP discovery and genotyping for evolutionary genetics using RAD sequencing Pp. 157–178 *in* Orgogozo V. and Rockman M. V., eds Molecular methods for evolutionary genetics. Humana Press, Totowa, NJ. 10.1007/978-1-61779-228-1_9 PMC3658458 22065437

[ece32072-bib-0020] Feulner, P. G. D. , F. J. J. Chain , M. Panchal , Y. Huang , C. Eizaguirre , M. Kalbe , et al. 2015 Genomics of divergence along a continuum of parapatric population differentiation. PLoS Genet. 11:e1004966. 25679225 10.1371/journal.pgen.1004966 PMC4334544

[ece32072-bib-0021] Foll, M. , and O. Gaggiotti . 2008 A genome‐scan method to identify selected loci appropriate for both dominant and codominant markers: a bayesian perspective. Genetics 180:977–993. 18780740 10.1534/genetics.108.092221 PMC2567396

[ece32072-bib-0022] Francis, R. C. , A. C. Havens , and M. A. Bell . 1985 Unusual lateral plate variation of threespine sticklebacks (*Gasterosteus aculeatus*) from Knik Lake, Alaska. Copeia 3:619–624.

[ece32072-bib-0023] Frichot, E. , S. D. Schoville , G. Bouchard , and O. Francois . 2013 Testing for associations between loci and environmental gradients using latent factor mixed models. Mol. Biol. Evol. 30:1687–1699. 23543094 10.1093/molbev/mst063 PMC3684853

[ece32072-bib-0024] Giles, N. 1983 The possible role of environmental calcium levels during the evolution of phenotypic diversity in outer hebridean populations of the three‐spined stickleback, *Gasterosteus aculeatus* . J. Zool. 199:535–544.

[ece32072-bib-0025] Grant, P. R. , and B. R. Grant . 2008 How and why species multiply. Princeton Univ. Press, Princeton.

[ece32072-bib-0026] Hagen, D. W. , and L. G. Gilbertson . 1972 Geographic variation and environmental selection in *Gasterosteus aculeatus* L. in the Pacific Northwest, America. Evolution 26:32. 10.1111/j.1558-5646.1972.tb00172.x 28555771

[ece32072-bib-0027] Hansson, T. , B. Fischer , A. B. Mazzarella , K. L. Voje , and L. A. Vøllestad . 2016 Lateral plate number in low‐plated threespine stickleback: a study of plasticity and heritability. Ecol. Evol., accepted article. 10.1002/ece3.2020 PMC4829041 27096076

[ece32072-bib-0028] Heuts, M. J. 1947 Experimental studies on adaptive evolution in *Gasterosteus aculeatus* L. Evolution 1:89–102.

[ece32072-bib-0029] Hohenlohe, P. A. , S. Bassham , P. D. Etter , N. Stiffler , E. A. Johnson , and W. A. Cresko 2010 Population genomics of parallel adaptation in threespine stickleback using sequenced RAD tags (DJ Begun, Ed,). PLoS Genet. 6:e1000862 20195501 10.1371/journal.pgen.1000862 PMC2829049

[ece32072-bib-0030] Jones, F. C. , Y. F. Chan , J. Schmutz , J. Grimwood , S. D. Brady , A. M. Southwick , et al. 2012a A genome‐wide SNP genotyping array reveals patterns of global and repeated species‐pair divergence in sticklebacks. Curr. Biol. 22:83–90. 22197244 10.1016/j.cub.2011.11.045 PMC3319444

[ece32072-bib-0031] Jones, F. C. , M. G. Grabherr , Y. F. Chan , P. Russell , E. Mauceli , J. Johnson , et al. 2012b The genomic basis of adaptive evolution in threespine sticklebacks. Nature 484:55–61. 22481358 10.1038/nature10944 PMC3322419

[ece32072-bib-0032] Kitano, J. , D. I. Bolnick , D. A. Beauchamp , M. M. Mazur , S. Mori , T. Nakano , et al. 2008 Reverse evolution of armor plates in the threespine stickleback. Curr. Biol. 18:769–774. 18485710 10.1016/j.cub.2008.04.027

[ece32072-bib-0033] Klepaker, T. 1993 Morphological changes in a marine population of threespined stickleback, *Gasterosteus aculeatus*, recently isolated in fresh water. Can. J. Zool. 71:1251–1258.

[ece32072-bib-0034] Klepaker, T. 1995 Postglacial evolution in lateral plate morphs in Norwegian freshwater populations of the threespine stickleback (*Gasterosteus aculeatus*). Can. J. Zool. 73:898–906.

[ece32072-bib-0035] Le Rouzic, A. , K. Østbye , T. Klepaker , T. Hansen , L. Bernatchez , D. Schluter , et al. 2011 Strong and consistent natural selection associated with armor reduction in sticklebacks. Mol. Ecol. 20:2483–2493. 21443674 10.1111/j.1365-294X.2011.05071.x

[ece32072-bib-0036] Lucek, K. , A. Sivasundar , and O. Seehausen . 2014 Disentangling the role of phenotypic plasticity and genetic divergence in contemporary ecotype formation during a biological invasion. Evolution 68:2619–2632. 24766190 10.1111/evo.12443

[ece32072-bib-0037] MacColl, A. D. C. , and B. Aucott . 2014 Inappropriate analysis does not reveal the ecological causes of evolution of stickleback armour: a critique of Spence *et al*. 2013. Ecol. Evol. 4:3509–3513. 25478143 10.1002/ece3.1179 PMC4224526

[ece32072-bib-0039] Mazzarella, A. B. , K. L. Voje , T. H. Hansson , A. Taugbøl , and B. Fischer . 2015 Strong and parallel salinity‐induced phenotypic plasticity in one generation of threespine stickleback. J. Evol. Biol. 28:667–677. 25656304 10.1111/jeb.12597

[ece32072-bib-0040] McKay, J. K. , and J. R. Stinchcombe . 2008 Ecological genomics of model eukaryotes. Evolution 62:2953–2957. 19055681 10.1111/j.1558-5646.2008.00536.x

[ece32072-bib-0042] Moodie, G. E. E. , and T. E. Reimchen . 1976 Phenetic variation and habitat differences in *Gasterosteus* populations of the Queen Charlotte Islands. Syst. Biol. 25:49–61.

[ece32072-bib-0043] O'Brown, N. M. , B. R. Summers , F. C. Jones , S. D. Brady , and D. M. Kingsley . 2015 A recurrent regulatory change underlying altered expression and Wnt response of the stickleback armor plates gene EDA. eLife, 4:e05290. 25629660 10.7554/eLife.05290 PMC4384742

[ece32072-bib-0044] Parmesan, C. , N. Ryrholm , C. Stefanescu , J. K. Hill , C. D. Thomas , H. Descimon , et al. 1999 Poleward shifts in geographical ranges of butterfly species associated with regional warming. Nature 399:579–583.

[ece32072-bib-0045] Pritchard, J. K. , J. K. Pickrell , and G. Coop . 2010 The genetics of human adaptation: hard sweeps, soft sweeps, and polygenic adaptation. Curr. Biol. 20:R208–R215. 20178769 10.1016/j.cub.2009.11.055 PMC2994553

[ece32072-bib-0046] Raj, A. , M. Stephens , and J. K. Pritchard . 2014 fastSTRUCTURE: variational inference of population structure in large SNP data sets. Genetics 197:573–589. 24700103 10.1534/genetics.114.164350 PMC4063916

[ece32072-bib-0047] Rambaut, A. , and A. J. Drummond . 2012 FigTree version 1.4. Available at: http://tree.bio.ed.ac.uk/software/figtree/. (accessed 19 May 2014).

[ece32072-bib-0048] Reimchen, T. E. 1983 Structural relationships between spines and lateral plates in threespine stickleback (*Gasterosteus aculeatus*). Evolution 37:931–946. 10.1111/j.1558-5646.1983.tb05622.x 28563533

[ece32072-bib-0049] Reimchen, T. E. 1984 Status of unarmoured and spine‐deficient populations (Charlotte unarmoured stickleback) of threespine stickleback, *Gasterosteus* sp., on the Queen Charlotte Islands, British Columbia. Can. Field Nat. 98:120–126.

[ece32072-bib-0050] Reimchen, T. E. 1992 Injuries on stickleback from attacks by a toothed predator (*Oncorhynchus*) and implications for the evolution of lateral plates. Evolution 46:1224. 10.1111/j.1558-5646.1992.tb00631.x 28564400

[ece32072-bib-0051] Rennison, D. J. , K. Heilbron , D. H. Barrett , and D. Schluter . 2015 Discriminating selection on lateral plate phenotype and its underlying gene, Ectodysplasin, in threespine stickleback. Am. Nat. 185:150–156. 25560560 10.1086/679280

[ece32072-bib-0052] Rockman, M. V. 2012 The QTN program and the alleles that matter for evolution: all that's gold does not glitter. Evolution 66:1–17. 22220860 10.1111/j.1558-5646.2011.01486.x PMC3386609

[ece32072-bib-0053] Schlichting, C. D. , and M. Pigliucci . 1998 Phenotypic evolution: a reaction norm perspective. Sinauer Associates Incorporated, Sunderland, MA.

[ece32072-bib-0054] Smith, G. , C. Smith , J. G. Kenny , R. R. Chaudhuri , and M. G. Ritchie . 2015 Genome‐wide DNA methylation patterns in wild samples of two morphotypes of threespine stickleback (*Gasterosteus aculeatus*). Mol. Biol. Evol. 32:888–895. 25534027 10.1093/molbev/msu344

[ece32072-bib-0055] Spence, R. , R. J. Wootton , I. Barber , M. Przybylski , and C. Smith . 2013 Ecological causes of morphological evolution in the three‐spined stickleback. Ecol. Evol. 3:1717–1726. 23789080 10.1002/ece3.581 PMC3686204

[ece32072-bib-0056] Stamatakis, A. 2006 Phylogenetic models of rate heterogeneity: a high performance computing perspective in Parallel and Distributed Processing Symposium 2006. IPDPS 2006. 20th International. IEEE.

[ece32072-bib-0057] Stamatakis, A. P. , H. Meier , and T. Ludwig . 2008 RAxML: a parallel program for phylogenetic tree inference. Available at: http://sco.h-its.org/exelixis/software.html

[ece32072-bib-0058] Svanbäck, R. , and D. Schluter . 2012 Niche specialization influences adaptive phenotypic plasticity in the threespine stickleback. Am. Nat. 180:50–59. 22673650 10.1086/666000

[ece32072-bib-0059] Teshima, K. M. , G. Coop , and M. Przeworski . 2006 How reliable are empirical genomic scans for selective sweeps? Genome Res. 16:702–712. 16687733 10.1101/gr.5105206 PMC1473181

[ece32072-bib-0060] Trucchi, E. , A. B. Mazzarella , G. D. Gilfillan , M. L. Romero , P. Schönswetter , and O. Paun . 2016 BsRADseq: screening DNA methylation in natural populations of non‐model species. Mol. Ecol., accepted article. doi: 10.1111/mec.13550 10.1111/mec.13550 PMC4949719 26818626

[ece32072-bib-0061] de Villemereuil, P. , E. Frichot , É. Bazin , O. Francois , and O. E. Gaggiotti . 2014 Genome scan methods against more complex models: when and how much should we trust them? Mol. Ecol. 23:2006–2019. 24611968 10.1111/mec.12705

[ece32072-bib-0062] Voje, K. L. , A. B. Mazzarella , T. F. Hansen , K. Østbye , T. Klepaker , A. Bass , et al. 2013 Adaptation and constraint in a stickleback radiation. J. Evol. Biol. 26:2396–2414. 24118552 10.1111/jeb.12240

[ece32072-bib-0063] Walker, J. A. , C. K. Ghalambor , O. L. Griset , D. McKenney , and D. N. Reznick . 2005 Do faster starts increase the probability of evading predators? Funct. Ecol. 19:808–815.

[ece32072-bib-0064] Ward, L. D. , and M. Kellis . 2012 Interpreting noncoding genetic variation in complex traits and human disease. Nat. Biotechnol. 30:1095–1106. 23138309 10.1038/nbt.2422 PMC3703467

[ece32072-bib-0065] West‐Eberhard, M. J. 1989 Phenotypic plasticity and the origins of diversity. Annu. Rev. Ecol. Syst. 20:249–278.

[ece32072-bib-0066] Wu, T. D. , and S. Nacu . 2010 Fast and SNP‐tolerant detection of complex variants and splicing in short reads. Bioinformatics 26:873–881. 20147302 10.1093/bioinformatics/btq057 PMC2844994

[ece32072-bib-0067] Wund, M. A. , J. A. Baker , B. Clancy , J. L. Golub , and S. A. Foster . 2008 A test of the “flexible stem” model of evolution: ancestral plasticity, genetic accommodation, and morphological divergence in the threespine stickleback radiation. Am. Nat. 172:449–462. 18729721 10.1086/590966

[ece32072-bib-0068] Wund, M. A. , S. Valena , S. Wood , and J. A. Baker . 2012 Ancestral plasticity and allometry in threespine stickleback reveal phenotypes associated with derived, freshwater ecotypes. Biol. J. Linn. Soc. 105:573–583. 10.5061/dryad.hb824gd4 PMC3351840 22611287

[ece32072-bib-0069] Yang, J. , B. Benjamin , B. P. McEnvoy , S. Gordon , A. K. Henders , D. R. Nyholt , et al. 2010 Common SNPs explain a large proportion of the heritability for human height. Nat. Genet. 42:565–569. 20562875 10.1038/ng.608 PMC3232052

[ece32072-bib-0070] Yoder, J. B. , E. Clancey , S. Des Roches , J. M. Eastman , L. Gentry , W. Godsoe , et al. 2010 Ecological opportunity and the origin of adaptive radiations. J. Evol. Biol. 23:1581–1596. 20561138 10.1111/j.1420-9101.2010.02029.x

