# Supplementary material for: Genomic signatures of the plateless phenotype in the threespine stickleback
Source: Ecol Evol. 2016 Apr 6;6(10):3161–73. doi: 10.1002/ece3.2072 (PMC4829042; doi:10.1002/ece3.2072)
Supplement: Supplementary file 1 — Appendix S1. Figure S1. (A) Average number of SNPs by position across loci for all reads in all libraries showing a very slight, nonsignificant increase after base 92. (B) Mean GC content across loci, showing a normal distribution. Figure S2. Genetic clustering analysis using FastStructure on the Melavatn population with K = 2. Table S1. Sample information: Fish # refers to the number of the fish within a Code. Table S2. Comma Separated Values containing 34 outlier loci (Table 1) and their genotype for all freshwater individuals included in the LFMM analysis. Table S3. Gene ontology keywords ranked by abundance for the nearest gene(s) listed in Table 1. [file ECE3-6-3161-s001.docx]

**APPENDIX**

Mazzarella *et al*. 2016

**Supporting Figure 1.** a) Average number of SNPs by position across loci for all reads in all libraries showing a very slight, non-significant increase after base 92. b) Mean GC content across loci, showing a normal distribution.


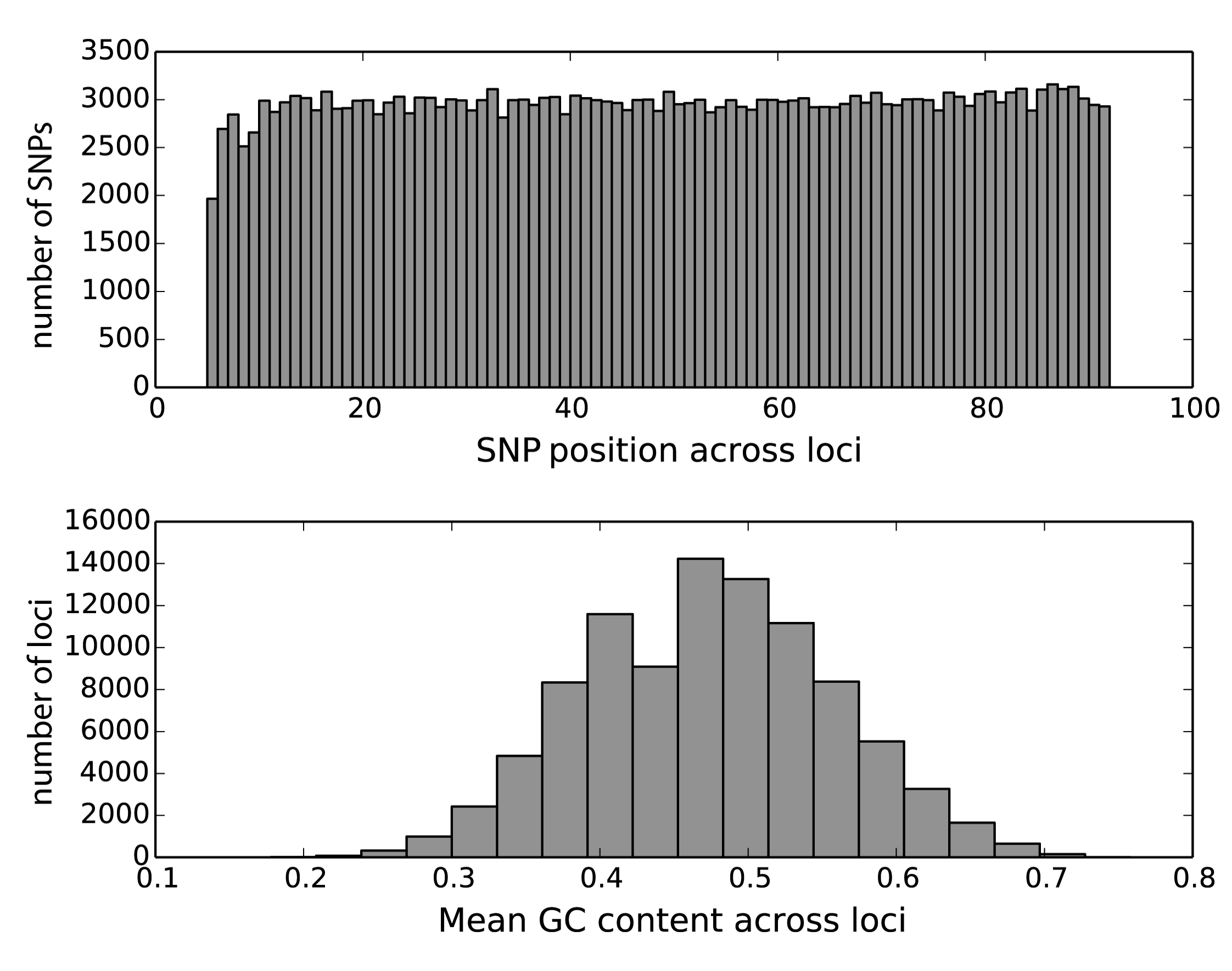


**Supporting Figure 2.** Genetic clustering analysis using FastStructure on the Melavatn population with K=2. L – Melavatn low plated, N – Melavatn plateless, with individual Fish Numbers corresponding to Supp. Table 1.

**Supporting Table 1.** Sample information: Fish # refers to the number of the fish within a Code. Code refers to a unique sampling attempt. Location refers to geographic location (as such there are groups with the same location, but a different code, indicating more than one sampling attempt at one location). RAD library indicates which of the 6 libraries each fish was sequenced in. Plate Morph indicates whether the fish is plateless, low plated, or full plated. Plate number is an actual plate count, performed for most freshwater fish. The next three columns indicate whether a particular individual was used for each analysis, the Sliding Window F_ST_ analysis, LFMM, and then RAxML, FastStructure and Bayescan (which were all run on the same set of individuals).

| **Code** | **Fish #** | **Location** | **RAD Library** | **Plate Morph** | **Plate #** | **F_ST_** | **LFMM** | **RAxML**  **FastStructure**  **Bayescan** |
| --- | --- | --- | --- | --- | --- | --- | --- | --- |
| MLN | 1 | Melavatn | 1 | plateless | 0 | x | x | x |
| MLN | 2 | Melavatn | 1 | plateless | 0 | x | x | x |
| MLN | 3 | Melavatn | 1 | plateless | 0 | x | x | x |
| MLN | 4 | Melavatn | 1 | plateless | 0 | x | x | x |
| MLN | 5 | Melavatn | 1 | plateless | 0 | x | x | x |
| MLN | 6 | Melavatn | 1 | plateless | 0 | x | x | x |
| MLN | 7 | Melavatn | 1 | plateless | 0 | x | x | x |
| MLN | 8 | Melavatn | 1 | plateless | 0 | x | x | x |
| MLN | 9 | Melavatn | 1 | plateless | n/a | x |  |  |
| MLN | 10 | Melavatn | 1 | plateless | n/a | x |  |  |
| MLN | 11 | Melavatn | 1 | plateless | n/a | x |  |  |
| MLL | 1 | Melavatn | 1 | low plated | 5 | x | x | x |
| MLL | 2 | Melavatn | 1 | low plated | 4 | x | x | x |
| MLL | 3 | Melavatn | 1 | low plated | 7 | x | x | x |
| MLL | 4 | Melavatn | 1 | low plated | 5 | x | x | x |
| MLL | 5 | Melavatn | 1 | low plated | 4 | x | x | x |
| MLL | 6 | Melavatn | 1 | low plated | n/a | x |  | x |
| MLL | 7 | Melavatn | 1 | low plated | 6 | x | x | x |
| MLL | 8 | Melavatn | 1 | low plated | 5 | x | x | x |
| MLL | 9 | Melavatn | 1 | low plated | 4 | x | x | x |
| MLL | 10 | Melavatn | 1 | low plated | n/a | x |  |  |
| MLL | 11 | Melavatn | 1 | low plated | n/a | x |  |  |
| MON | 5 | Mosvatn | 2 | plateless | n/a | x |  |  |
| MON | 6 | Mosvatn | 2 | plateless | n/a | x |  |  |
| MON | 7 | Mosvatn | 2 | plateless | 0 | x | x | x |
| MON | 8 | Mosvatn | 2 | plateless | 0 | x | x | x |
| MON | 9 | Mosvatn | 2 | plateless | 0 | x | x | x |
| MON | 10 | Mosvatn | 2 | plateless | 0 | x | x | x |
| MON | 11 | Mosvatn | 2 | plateless | 0 | x | x | x |
| MON | 12 | Mosvatn | 2 | plateless | 0 | x | x |  |
| MON | 13 | Mosvatn | 2 | plateless | 0 | x | x | x |
| MON | 14 | Mosvatn | 2 | plateless | 0 | x | x | x |
| MON | 15 | Mosvatn | 2 | plateless | 0 | x | x | x |
| MON | 16 | Mosvatn | 2 | plateless | 0 | x | x | x |
| MON | 17 | Mosvatn | 2 | plateless | 0 | x | x | x |
| MON | 18 | Mosvatn | 2 | plateless | 0 | x | x | x |
| MON | 19 | Mosvatn | 2 | plateless | 0 | x | x | x |
| MON | 20 | Mosvatn | 2 | plateless | 0 | x | x | x |
| MOL | 1 | Mosvatn | 2 | low plated | 3 | x | x | x |
| MOL | 2 | Mosvatn | 2 | low plated | 5 | x | x | x |
| MOL | 3 | Mosvatn | 2 | low plated | n/a | x |  | x |
| MOL | 4 | Mosvatn | 2 | low plated | 6 | x | x | x |
| MOL | 5 | Mosvatn | 2 | low plated | 4 | x | x | x |
| MOL | 6 | Mosvatn | 2 | low plated | 4 | x | x | x |
| MOL | 7 | Mosvatn | 2 | low plated | 3 | x | x | x |
| MOL | 8 | Mosvatn | 2 | low plated | n/a | x |  | x |
| MOL | 9 | Mosvatn | 4 | low plated | n/a | x |  |  |
| MOL | 10 | Mosvatn | 4 | low plated | n/a | x |  |  |
| BAN | 1 | Bårdsrudtjern | 3 | plateless | 0 | x | x | x |
| BAN | 2 | Bårdsrudtjern | 3 | plateless | n/a | x |  |  |
| BAN | 3 | Bårdsrudtjern | 3 | plateless | n/a | x |  |  |
| BAN | 4 | Bårdsrudtjern | 3 | plateless | 0 | x | x | x |
| BAN | 5 | Bårdsrudtjern | 4 | plateless | 0 | x | x | x |
| BAN | 6 | Bårdsrudtjern | 4 | plateless | 0 | x | x | x |
| BAN | 7 | Bårdsrudtjern | 4 | plateless | 0 | x | x | x |
| BAN | 8 | Bårdsrudtjern | 4 | plateless | 0 | x | x | x |
| BAN | 9 | Bårdsrudtjern | 4 | plateless | 0 | x | x | x |
| BAN | 10 | Bårdsrudtjern | 4 | plateless | 0 | x | x | x |
| BAL | 1 | Bårdsrudtjern | 3 | low plated | 4 | x | x | x |
| BAL | 2 | Bårdsrudtjern | 3 | low plated | 4 | x | x | x |
| BAL | 3 | Bårdsrudtjern | 3 | low plated | 6 | x | x | x |
| BAL | 4 | Bårdsrudtjern | 3 | low plated | 2 | x | x | x |
| BAL | 5 | Bårdsrudtjern | 3 | low plated | 2 | x | x | x |
| BAL | 6 | Bårdsrudtjern | 3 | low plated | 8 | x | x | x |
| BAL | 7 | Bårdsrudtjern | 3 | low plated | 2 | x | x | x |
| BAL | 8 | Bårdsrudtjern | 3 | low plated | 7 | x | x | x |
| BAL | 9 | Bårdsrudtjern | 4 | low plated | 6 | x | x | x |
| BAL | 10 | Bårdsrudtjern | 4 | low plated | 6 | x | x | x |
| DO | 1 | Drøbak Marine | 1 | Fully plated | n/a | x |  |  |
| DO | 2 | Drøbak Marine | 1 | Fully plated | n/a | x |  |  |
| DO | 3 | Drøbak Marine | 1 | Fully plated | n/a | x |  |  |
| DO | 4 | Drøbak Marine | 1 | Fully plated | n/a | x |  |  |
| DO | 5 | Drøbak Marine | 1 | Fully plated | n/a | x |  |  |
| DO | 6 | Drøbak Marine | 1 | Fully plated | n/a | x |  |  |
| DO | 11 | Drøbak Marine | 5 | Fully plated | n/a | x |  | x |
| DO | 13 | Drøbak Marine | 6 | Fully plated | n/a | x |  | x |
| DS | 1 | Drøbak Marine | 3 | Fully plated | n/a | x |  |  |
| DS | 3 | Drøbak Marine | 3 | Fully plated | n/a | x |  | x |
| DS | 5 | Drøbak Marine | 4 | Fully plated | n/a | x |  | x |
| DS | 6 | Drøbak Marine | 4 | Fully plated | n/a | x |  | x |
| DS | 8 | Drøbak Marine | 5 | Fully plated | n/a | x |  |  |
| DS | 9 | Drøbak Marine | 5 | Fully plated | n/a | x |  |  |
| DS | 17 | Drøbak Marine | 6 | Fully plated | n/a | x |  |  |
| DS | 22 | Drøbak Marine | 3 | Fully plated | n/a | x |  |  |
| DS | 23 | Drøbak Marine | 3 | Fully plated | n/a | x |  |  |
| DS | 24 | Drøbak Marine | 4 | Fully plated | n/a | x |  |  |
| DS | 25 | Drøbak Marine | 4 | Fully plated | n/a | x |  |  |
| DS | 26 | Drøbak Marine | 5 | Fully plated | n/a | x |  | x |
| BR | 2 | Bergen Marine | 6 | Fully plated | n/a | x |  |  |
| BR | 4 | Bergen Marine | 3 | Fully plated | n/a | x |  |  |
| BR | 5 | Bergen Marine | 3 | Fully plated | n/a | x |  |  |
| BR | 6 | Bergen Marine | 4 | Fully plated | n/a | x |  |  |
| BR | 7 | Bergen Marine | 4 | Fully plated | n/a | x |  |  |
| BR | 8 | Bergen Marine | 5 | Fully plated | n/a | x |  |  |
| BR | 9 | Bergen Marine | 5 | Fully plated | n/a | x |  |  |
| BR | 10 | Bergen Marine | 5 | Fully plated | n/a | x |  | x |
| BR | 11 | Bergen Marine | 6 | Fully plated | n/a | x |  |  |
| BR | 12 | Bergen Marine | 6 | Fully plated | n/a | x |  |  |
| BR | 14 | Bergen Marine | 6 | Fully plated | n/a | x |  |  |
| BR | 16 | Bergen Marine | 3 | Fully plated | n/a | x |  |  |
| BR | 17 | Bergen Marine | 3 | Fully plated | n/a | x |  |  |
| BR | 18 | Bergen Marine | 4 | Fully plated | n/a | x |  |  |
| BR | 19 | Bergen Marine | 4 | Fully plated | n/a | x |  |  |
| BR | 20 | Bergen Marine | 5 | Fully plated | n/a | x |  | x |
| BR | 21 | Bergen Marine | 5 | Fully plated | n/a | x |  |  |
| BR | 22 | Bergen Marine | 5 | Fully plated | n/a | x |  |  |
| BR | 23 | Bergen Marine | 6 | Fully plated | n/a | x |  |  |
| BR | 24 | Bergen Marine | 6 | Fully plated | n/a | x |  | x |
| FL | 1 | Flødevigen Marine | 6 | Fully plated | n/a | x |  | x |
| FL | 3 | Flødevigen Marine | 3 | Fully plated | n/a | x |  |  |
| FL | 4 | Flødevigen Marine | 3 | Fully plated | n/a | x |  |  |
| FL | 5 | Flødevigen Marine | 4 | Fully plated | n/a | x |  |  |
| FL | 6 | Flødevigen Marine | 4 | Fully plated | n/a | x |  |  |
| FL | 7 | Flødevigen Marine | 5 | Fully plated | n/a | x |  |  |
| FL | 8 | Flødevigen Marine | 5 | Fully plated | n/a | x |  | x |
| FL | 9 | Flødevigen Marine | 5 | Fully plated | n/a | x |  | x |
| FL | 10 | Flødevigen Marine | 6 | Fully plated | n/a | x |  |  |
| FL | 11 | Flødevigen Marine | 6 | Fully plated | n/a | x |  | x |
| FL | 12 | Flødevigen Marine | 6 | Fully plated | n/a | x |  | x |
| FL | 15 | Flødevigen Marine | 3 | Fully plated | n/a | x |  |  |
| FL | 16 | Flødevigen Marine | 3 | Fully plated | n/a | x |  |  |
| FL | 17 | Flødevigen Marine | 4 | Fully plated | n/a | x |  |  |
| FL | 18 | Flødevigen Marine | 4 | Fully plated | n/a | x |  |  |
| FL | 19 | Flødevigen Marine | 5 | Fully plated | n/a | x |  | x |
| FL | 20 | Flødevigen Marine | 5 | Fully plated | n/a | x |  |  |
| FL | 21 | Flødevigen Marine | 5 | Fully plated | n/a | x |  |  |
| FL | 23 | Flødevigen Marine | 6 | Fully plated | n/a | x |  | x |
| FL | 25 | Flødevigen Marine | 6 | Fully plated | n/a | x |  | x |

**Supporting Table 3.** Gene ontology keywords ranked by abundance for the nearest gene(s) listed in table 1.

| **GO term** | **# of genes listed** |
| --- | --- |
| Protein binding (molecular function) | 7 |
| Membrane (cellular component) | 3 |
| Sequence-specific DNA binding (molecular function, DNA binding) | 3 |
| Anatomical structure development | 2 |
| Apoptotic process (cell death, biological process) | 2 |
| ATP binding | 2 |
| Intracellular (cellular component, cell) | 2 |
| Nucleotide binding | 2 |
| Posterior lateral line neuromast primordium migration (locomotion, cell motility, anatomical structure development, biological process) | 2 |
| Regulation of DNA methylation (biological process) | 2 |
| Regulation of transcription, DNA-Dependent (biological process) | 2 |
| Sequence-specific DNA binding transcription factor activity (molecular function, nucleic acid binding transcription factor activity) | 2 |
| Skeletal system development (anatomical structure development, biological process) | 2 |
